# Abnormalities in the meibomian glands in patients with oral administration of anticancer combination drug-capsule TS-1^®^: a case report

**DOI:** 10.1186/s12885-015-1781-0

**Published:** 2015-10-24

**Authors:** Shin Mizoguchi, Yuka Okada, Masahide Kokado, Shizuya Saika

**Affiliations:** Department of Ophthalmology, Wakayama Medical University School of Medicine, 811-1 Kimiidera, Wakayama, 641-0012 Japan

**Keywords:** Meibomian gland, Meibography, TS-1^®^

## Abstract

**Background:**

The anticancer TS-1^®^ combination capsules of tegafur, gimeracil, and oteracil potassium (Taiho Pharmaceutical Co. Ltd, Japan) causes side effects, *i. e.,* corneal epithelial disorder and dacryostenosis. However, its side effect on meibomian gland had not been reported. We observed morphological changes in the meibomian gland in patients taking TS-1^®^ who exhibited punctate corneal epithelial defects to examine if dysfunction of meibomian glands is involved in the corneal epitheliopathy.

**Case presentation:**

Patients comprised two males and one female (age, 59–81 years). After starting oral TS-1^®^ administration, patients developed subjective symptoms such as decreased visual acuity. Corneal epithelial disorder was seen in all six eyes of the three subjects exhibited, and lacrimal duct disorder was seen in one eye. Furthermore, meibomian gland loss and contraction were observed in all six eyes that exhibited meibomian gland disorder upon examination by using the MeiboPen^®^.

**Conclusions:**

Results suggested that oral administration of TS-1^®^ may cause meibomian gland disorder which potentially affect corneal epithelial homeostasis.

## Background

An anticancer combination capsules, TS-1^®^ combination capsules (Taiho Pharmaceutical Co. Ltd, Japan), contain tegafur, a 5-fluorouracil (5-FU) prodrug; gimeracil, a 5-FU decomposing enzyme inhibitor; and oteracil potassium, which blocks 5-FU activation in the digestive tract, thereby inhibiting the development of gastrointestinal disorder. TS-1^®^ is currently used for a wide range of malignant tumors, including gastric cancer, rectal cancer, head and neck cancer, and lung cancer [1, 2].

5-FU is incorporated into RNA as a substitute for uracil. The 5-FU metabolite fluorodeoxyuridine monophosphate blocks DNA synthesis [3]. 5-FU exhibits anti-tumor effects via these inhibitions of RNA function and DNA synthesis; however, since it is also incorporated into healthy cells along with tumor cells, it often causes side effects in tissues with actively dividing cells [4].

Reported major side effects in ocular tissues include corneal epithelial disorder, dacryoma, dacryostenosis, and lacrimal duct obstruction, which are caused by 5-FU transferred into lacrimal fluid. There are various types of corneal epithelial disorders, including superficial punctate keratopathy-like lesions and false branch-shaped lesions [5–7]. The existence of the lumen occlusive side effects of dacryoma, dacryostenosis, and lacrimal duct obstruction suggests that meibomian gland disorders could also occur. These findings suggest that lacrimal fluid containing 5-FU is secreted to the eye surface, causing inflammation around orifice of the meibomian gland. Such inflammation may subsequently have caused keratinization and fibrosis, leading to obstruction of the orifice of meibomian gland and resulting in meibomian gland obstruction and subsequent tissue damage. However, there have been no detailed investigations pertaining to the effects of TS-1^®^ on the meibomian glands, which are an appendage of the surface of the eye. We applied non-invasive mobile meibography (MeiboPen^®^; Japan Focus Company Ltd., Tokyo, Japan) to patients who had corneal epithelial disorders using TS-1^®^ to observe morphological changes in meibomian glands and assessed the results in three patients evaluated using meibo-scores [8, 9].

## Case presentation

### Case 1

**Fig. 1:**
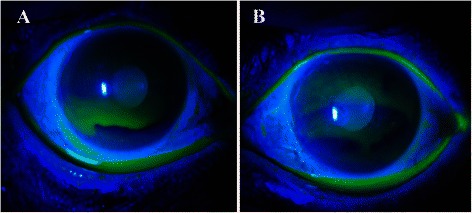
Case 1. Anterior segment photography. Superficial punctate keratopathy-like lesions observed in both eyes from the central to lower part of the cornea. **a**: right eye, **b**: left eye

**Fig. 2:**
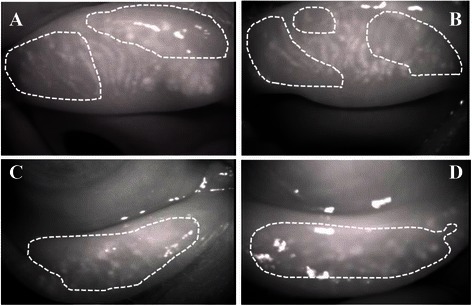
Case 1. Meibography. **a**, Upper right eyelid. Meibo score 3; **b**, Upper left eyelid. Meibo score: 3; **c**, Lower right eyelid. Meibo score: 3; **d**, Lower left eyelid. Meibo score: 3. Dotted lines indicate the loss and contraction of meibomian glands zone

A 59-year-old male patient underwent surgery for gallbladder cancer in June 2011. He was administered oral TS-1^®^ (100 mg/day) from August 2011, and after about 3 months, he started experiencing discomfort in both eyes; he was examined at our department in July 2012. He did not receive systemic and topical medications that might cause tissue damage in the ocular surface. His visual acuity was 0.5 (0.8) in the right eye and 0.6 (0.8) in the left eye. Intraocular pressure was 10 mmHg in both eyes. Schirmer I test (with topical oxybuprocaine hydrochloride) did not show reduction of tear secretion (26 mm for the right eye and 35 mm for the left eye). Slit-lamp microscopy indicated punctate keratopathy-like lesions in the cornea of both eyes (Fig. 1a, b). No lacrimal punctum obstruction or lacrimal duct obstruction were observed. Observation of the meibomian glands with the MeiboPen^®^ revealed loss and contraction of meibomian glands (white dotted line range). Meibo-score was 6 in both eyes. While administration of TS-1^®^ was continued, diquafosol sodium eye drops were also prescribed, and the patient’s course was observed. Consequently, both corneas improved, as did the patient’s subjective symptoms (Fig. 2a, b, c, d).

### Case 2

**Fig. 3:**
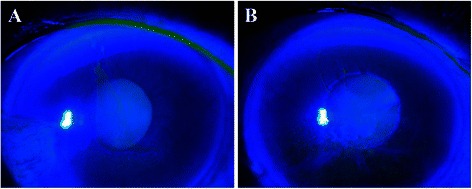
Case 2. Anterior segment photography. **a**, right eye, **b**; left eye. Epithelial defects were noted near the central part of the cornea in both eyes

**Fig. 4:**
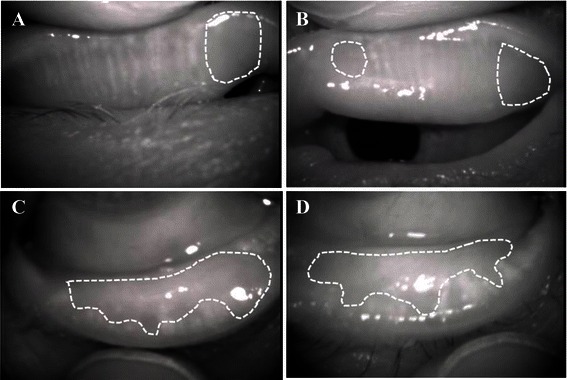
Case 2. Meibography. **a**, Upper right eyelid. Meibo score: 1; **b**, Upper left eyelid. Meibo score: 1; **c**, Lower right eyelid. Meibo score: 3; **d**, Lower left eyelid. Meibo score: 3. Dotted lines indicate the loss and contraction of meibomian glands zone

An 81-year-old female underwent surgery for gingival cancer in May 2012. Postoperatively, oral administration of 80 mg/day of TS-1^®^ was initiated, following which she experienced blurred vision in both eyes and went to see her regular physician. She was diagnosed with corneal epithelial disorder and given additional treatment with sodium hyaluronate eye drops. However, the corneal epithelial disorder deteriorated, so she was referred to our department in January 2013. She had already discontinued TS-1^®^ treatment when we examined her. She did not receive systemic and topical medications that might cause tissue damage in the ocular surface. Her visual acuity was 0.06 (noncorrigunt) in the right eye and 0.04 (noncorrigunt) in the left eye. Intraocular pressure was 12 mmHg in the right eye and could not be measured in the left eye. Schirmer I test (with topical oxybuprocaine hydrochloride) showed reduction of tear secretion (0 mm for both eyes). Slit-lamp microscopy indicated corneal erosion near the central part of the cornea in both eyes (Fig. 3a, b). No lacrimal punctum obstruction or dacryostenosis were noted in either eye. Loss and contraction of meibomian glands were observed. Meibo score was 4 in the right eye and 4 in the left eye (Fig. 4a, b, c, d). Rebamipide ophthalmic solution and ofloxacin eye ointment were additionally prescribed and the patient was followed up regularly. Consequently, the corneal condition improved, as did the patient’s subjective symptoms.

### Case 3

A 66-year-old male underwent surgery for gallbladder cancer in July 2012. Oral administration of TS-1^®^ (80 mg/day) was initiated in August 2012, and approximately 2 months later, the patient experienced decreased visual acuity in both eyes; he was examined at our department in January 2013. He did not receive systemic and topical medications that might cause tissue damage in the ocular surface. His visual acuity was 0.2 (0.3) in the right eye and 0.15 (0.2) in the left eye. Intraocular pressure was 20 mmHg in the right eye and 11 mmHg in the left eye. Schirmer I test (with topical oxybuprocaine hydrochloride) showed reduction of tear secretion (21 mm for the right eye and 8 mm for the left eye). Slit-lamp microscopy indicated superficial punctate keratopathy-like lesions in the corneas of both eyes. Lacrimal punctum narrowing and lacrimal duct obstruction were observed in the right eye only. Loss and contraction of meibomian glands were observed, and meibo score was 4 for the right eye and 3 for the left eye. Funduscopy indicated simple diabetic retinopathy. However, TS-1^®^ administration was continued, and it was decided that the patient be regularly followed up by his local physician.

## Discussion

We observed meibomian gland loss and contraction in all six eyes of patients taking TS-1^®^ whom we examined for meibomian gland disorders. Although meibo-score is known to increase with age, the mean meibo score in healthy individuals in their 50s to their 80s has been reported as < 2 [8]. Meibo-scores for our patients ranged 3 – 6. We thus consider that TS-1^®^ might have been responsible for meibomian gland abnormality. Corneal epithelial disorder was observed in all three cases (six eyes) examined in this study, and lacrimal duct disorder was noted in one eye. The possible reason why the degree of the damage differed aming each gland is to be clarified. However, explanation might include the size the of the duct affects the obstruction of the orifice of the gland.

A previous report of a pathological examination showed that repeated administration of TS-1^®^ induced focal necrosis, epithelial hyperplasia and neutrophilic inflammation in conjunctival in dogs [10]. Incresead concentration of 5-FU upon systemic administration of TS-1^®^ causes obstruction of lacrymal apparatus [7]. These reports suggest that 5-FU in tear fluid might induce local inflammation in/around orifice of the glands. This inflammation may subsequently have caused keratinization and fibrosis, leading to obstruction of meibomian gland orifice and resulting in meibomian gland obstruction and loss. A manuscript is available that report on the meibomian gland abnormality in patients with longstanding wearing orbital prosthesis, suggesting that conjunctival persistent subclinical inflammation might cause the phenomenon [11]. This report also support the possibility that TS-1^®^ might damage the orifice of the meibomian gland.

Function of meibomian glands is considered to depend on proliferation of its acinar cells. Cell proliferation is mediated a set of variety of enzymes including thymidylate synthase of which expression is upregulated by various growth factors. 5-FU is known to inhibit the activity of this enzyme, leading to attenuation of cell growth. It is to be examined if systemic TS-1^®^ affects cell proliferation activity in meibomian glands in animal experiments.

Treatment may include (i) TS-1^®^ discontinuation, (ii) frequent use of artificial lacrimal fluid and dry eye treatment with sodium hyaluronate eye drops to wash out TS-1^®^ in lacrimal fluid and protect the corneas, (iii) use of antibiotics to prevent infection in cases of corneal epithelium loss, and (iv) if dacryostenosis is suspected, lacrimal punctum incision or silicon tube placement. It has also been reported that symptoms improved in cases of persistent corneal epithelial disorder persists after cessation of TS-1^®^. Topical treatment for the damage in the corneal epithelium in Cases 1 and 2 was considered to be effective presumably due to direct effects on the corneal epithelial homeostasis, but not on the meibomian glands. Administration with autologous serum eye drops [12], a hot compress, a conventional treatment for meibomian gland dysfunction, and lid hygiene may not only improve meibomian gland function, but also alleviate symptoms of corneal epithelial disorder.

In the current manuscript we report meibomian gland abnormality in patients with TS-1^®^ administration. The finding here prompts us to plan not only a retrospective survey but also a prospective study to examine the effects of TS-1^®^ on meibomian gland morphology and function.

## Conclusions

Oral administration of TS-1^®^ might cause meibomian gland disorder which potentially affect corneal epithelial homeostasis. Further study is needed to uncover the side effects of TS-1^®^ on meibomian gland morphology and function.

## Consent

This study was performed in accordance with the Helsinki Declaration. Written informed consent were obtained from all three patients for publication of this Case report and any accompanying images. The copys of the written consent are available for review by the Editor of this journal.

## Abbreviation

5-FU: 5-fluorouracil
